# Gene expression profiling of SPIN1 in gastric cancer: insights into tumorigenesis and potential therapeutic targets

**DOI:** 10.3389/fgene.2025.1510849

**Published:** 2025-06-11

**Authors:** Bei-Bei Lv, Lei Cai, Yao Xiao, Rui-Han Wang, Xiao-Yan Lin

**Affiliations:** Department of Pathology, Shandong Provincial Hospital Affiliated To Shandong First Medical University, Jinan, Shandong, China

**Keywords:** gastric cancer, SPIN1, prognosis, immune microenvironment, m6A and m5C associated genes

## Abstract

**Background:**

Gastric cancer (GC) is a prevalent malignant tumor globally, posing a significant threat to human health. The histone code reader Spindlin1 (SPIN1) has been implicated in tumorigenesis and tumor progression, however, the exact molecular mechanisms underlying these processes remain incompletely understood. The biological function and regulatory mechanisms of SPIN1 in GC remain ambiguous. This study aims to investigate the regulatory mechanisms of SPIN1 in the pathogenesis and progression of GC, as well as to identify genes closely associated with SPIN1 and potential biomarkers.

**Methods:**

Gene expression profiles from 375 patients diagnosed with gastric cancer (GC) and 32 control subjects were obtained from the TCGA-STAD database. Our study examined the relationships between SPIN1 expression and various factors including tumor progression, clinical stage, survival status, immune microenvironment and drug sensitivity within the cohort of 375 GC patients and 32 controls. Furthermore, we investigated the interplay between m6A and 5 mC regulators in influencing the expression of SPIN1 in GC, and identified genes with significant correlations with SPIN1 through Spearman correlation analysis.

**Results:**

A significantly elevated expression of SPIN1 was found in 375 GC patients compared to 32 control subjects. SPIN1 expression was positively correlated with EMT score and angiogenesis score. Cell proliferation-related gene sets (myogenesis, mitotic spindle and G2M checkpoint) were all significantly associated with the high SPIN1 GC group. Eosinophils was associated with high expression of SPIN1. A total of 21 checkpoints were associated with SPIN1 expression. Low SPIN1 expression group could benefit from Axitinib, Cytarabine, Pazopanib and Sunitinib. Most m6A regulators and a subset of m5C regulators were positively associated with SPIN1. Finally, we screened the 10 genes with the strongest correlation with SPIN1, among which CDH11 and SLC8A1 were associated with the prognosis of GC.

**Conclusion:**

In conclusion, our study has provided valuable insights into the pivotal role of SPIN1 in GC development, elucidating its potential molecular mechanisms and establishing it as a promising therapeutic target.

## 1 Introduction

Gastric cancer (GC), one of the most prevalent malignant tumors worldwide, ranks as the third leading cause of cancer-related mortality (Sung et al., 2021). Due to the significant heterogeneity and diverse histological manifestations of GC, systemic therapeutic advancements have been limited in recent years, while targeted therapies have gained prominence in the management of advanced GC. Consequently, it is imperative to identify genetic alterations that are both sensitive and specific to GC development, and to investigate the molecular mechanisms underlying their roles, which are of considerable theoretical and practical importance.

SPIN1, a member of the Spin/Ssty family, was initially identified as a maternal transcript expressed during the transition from oocytes to embryos in mice (Oh et al., 1997). As a high-affinity reader of histone modifications, SPIN1 comprises three Tudor-like domains and facilitates rRNA expression by binding to H3K4me3, thereby influencing epigenetic regulation (Wang et al., 2011; Zhang et al., 2018). Given that the expression of m6A methyltransferases (e.g., METTL3) and m5C methyltransferases (e.g., NSUN2) is associated with H3K4me3 modifications (Xiong et al., 2022; Chen et al., 2024), we hypothesize that SPIN1 may interact with METTL3 and NSUN2 through epigenetic regulatory mechanisms, potentially mediating crosstalk between histone modifications and RNA methylation pathways.

SPIN1 was shown to be highly expressed in various types of malignant tumors, including ovarian cancer, breast cancer, non-small cell lung cancer, liver cancer, and liposarcoma (Zhou et al., 2022; Jin et al., 2021; Wang L. et al., 2020; Zhao et al., 2020). Our preliminary investigations have revealed elevated expression of SPIN1 in GC tissues, closely correlating with unfavorable prognoses for patients. SPIN1 enhances the proliferation, migration, and invasion of GC cells. By binding to H3K4me3, SPIN1 activates the MDM2-p21-E2F1 signaling pathway, thereby promoting GC cell proliferation (Lv et al., 2020). Nevertheless, the full extent of SPIN1’s impact on prognosis and treatment outcomes in GC remains ambiguous. The mechanistic role of SPIN1 in GC warrants further exploration. Consequently, this study aims to delve deeper into the molecular characteristics of SPIN1 in GC and to identify genes associated with its function, thereby providing a comprehensive understanding of its multifaceted role.

## 2 Materials and methods

### 2.1 Data source

Generally, all the microarray data after normalization were analyzed by R software.

We collected the gene expression data in the database of The Cancer Genome Atlas Program (TCGA-STAD, https://portal.gdc.cancer.gov). The research included the data of 375 GC tumor samples and 32 normal samples. Clinical information of GC patients was downloaded from TCGA-STAD dataset, including age, gender, survival status, grade, tumor stage and TNM stage. To ensure the similarity of the two groups of samples in key characteristics, we used the compareGroups package (Version: 4.9.1) to draw the table of clinical baseline characteristics. In addition, the ACRG/GSE62254 datasets comprised of transcriptomic expression profiling and correspondingly clinical information derived from Asian cohorts and downloaded from Gene Expression Ominibus (GEO)(https://www.ncbi.nlm.nih.gov/geo/query).

### 2.2 SPIN1 expression analysis of GC

Gene raw expression data from TCGA were normalized, and Ensemble ID from TCGA were converted to gene symbol. The expression of SPIN1 was extracted from TCGA dataset and compared in tumor and normal groups. The Kruskal–Wallis test was used to analyze the relationship between SPIN1 and the clinical characteristics of GC.

### 2.3 Gene set variation analysis

GSVA is a method to estimate variation of gene set enrichment through the samples of expression data set. The “GSVA” R package was used to find the pathway associated with SPIN1 expression. Here, we used GSVA analysis to calculate the enrichment scores of metastasis-related pathways in GC samples including EMT score and angiogenesis score. The pathway of EMT and angiogenesis were obtained from HALLMARK_EPITHELIAL_MESENCHYMAL_ TRANSITION and HALLMARK_ANGIOGENESIS in HALLMARK database. The adj.p-value <0.05 was regarded as statistically significant.

### 2.4 Gene set enrichment analysis

To investigate the biological functions of SPIN1, we performed single-gene Gene Set Enrichment Analysis (GSEA)(adj.p < 0.05) using the R package “clusterProfiler” (v 4.8.3) (Wu et al., 2021), with the background gene sets (h.all.v2023.2.Hs.symbols.gmt) downloaded from the GSEA database (http://www.gsea-msigdb.org/gsea/msigdb). In addition, the analysis dataset was partitioned into two subgroups - SPIN1-high and SPIN1-low - with the median expression value serving as the grouping threshold. GSEA (adj.p < 0.05) was performed using genes ranked by logFC values from differential expression analysis in descending order.

Besides, Gene Set Variation Analysis (GSVA) was employed to evaluate pathway enrichment scores using the R packages “GSVA,” “limma,” and “GSEABase.” Data preprocessing, normalization, and statistical comparisons were conducted with the “limma” package to identify differentially enriched pathways. For downstream visualization, processed data were structured using the “data.table” package, and heatmaps were generated via the “gplots” package with color palettes optimized by the “RColorBrewer” package. Statistical significance was defined as a false discovery rate (FDR)-adjusted p-value <0.05.

### 2.5 STAD mutation data

Genetic mutation data for TCGA-STAD were obtained from the original MAF (Mutation Annotation Format) files. We screened mutated genes in high and low SPIN1 expression groups and compared TMB scores in high and low SPIN1 expression groups. The mutation landscape in the TCGA-STAD cohort was presented by the ‘maftools’ R package.

### 2.6 Evaluation of the immune microenvironment landscape and drug sensitivity analysis

The immune scores and stromal scores could predict the content of immune and stromal components in tumors. Immune and stromal scores of GC samples were calculated using the ESTIMATE algorithm (Yoshihara et al., 2013), which was provided in the R package “ESTIMATE”. Then we used CIBERSORT databases to screen the differential immune cells between high and low SPIN1 expression groups. Gene annotation was performed using the org.Hs.e.g.,.db database to convert gene ENTREZ IDs to SYMBOLs. Immune infiltration analysis was then conducted using the deconvo_cibersort function from the IOBR package. Furthermore, we analyzed the correlation between SPIN1 and 37 immune checkpoints, and the False Discovery Rate (FDR) method was adopted for multiple testing correction. Based on immune checkpoints significantly associated with SPIN1, the differences in immune checkpoint molecule expression levels between SPIN1-high and SPIN1-low groups were compared using the nonparametric Mann-Whitney U rank-sum test.

The drug response data were obtained from the Genomics of Drug Sensitivity in Cancer (GDSC) database. We predicted the sensitivity of each patient to chemotherapeutic agents by the R package “pRRophetic” (Geeleher et al., 2014). The estimated IC50 value for each patient treated with a specific chemotherapy drug was obtained through the function “pRRopheticPredict”. Specially, the gene expression datas were processed using the lc.tableToNum function, followed by batch correction with the eb method. Phenotypic data underwent power transformation, and genes with variation below the 0.2 threshold were filtered out. The correlation between predicted IC50 values and SPIN1 gene expression levels was analyzed using the cor.test function. The obtained P-values for correlations between multiple drugs and SPIN1 expression levels were subsequently adjusted for multiple hypothesis testing using the p.adjust function. Then, we calculated the correlation between SPIN1 expression and 7 chemotherapy drugs including 5-Fluorouracil, Axitinib, Cytarabine, Mitomycin C, Pazopanib, Sunitinib and Trametinib.

### 2.7 Correlations of SPIN1 with m6A and m5C associated genes

The R software package was utilized to evaluate the correlation between the expression of SPIN1 and the expression of 20 m6A modifiers (Luo et al., 2024), including “readers” ELAVL1, FMR1, YTHDC1, YTHDC2, IGF2BP1, IGF2BP2, YTHDF1, YTHDF2, YTHDF3, HNRNPA2B1, TRA2A, RBMX, “writers” METTL14, METTL3, RBM15, WTAP, ZC3H13, CBLL1, and “erasers” FTO, ALKBH5. In addition, the R software package was utilized to evaluate the correlation between the expression of SPIN1 and the expression of m5C regulators (Luo et al., 2024), including “writers” DNMT1, DNMT3A, and DNMT3B, “readers” MBD1, MBD2, MBD3, MBD4, MECP2, NEIL1, NTHL1, SMUG1, TDG, UHRF1, UHRF2, UNG, ZBTB38, ZBTB33, and ZBTB4, and “erasers” TET1, TET2, and TET3. And the FDR method was adopted for multiple testing correction. The data were analyzed visually by ggplot2 software package. In addition, boxplots were constructed to compare expression levels of METTL and NSUN2 between SPIN1-high and SPIN1-low groups, with group stratification determined by median SPIN1 expression. Inter-group differences were assessed using the Wilcoxon rank-sum test.

### 2.8 Screening of SPIN1 related genes in GC

R package “DEseq2” was used to identify differentially expressed genes (DEGs), between GC samples and controls using∣logFC | >1 along with P.adj.val <0.05 as the threshold (Ritchie et al., 2015).

Then we screened the high correlation genes with SPIN1 from DEGs. Correlations were assessed with Spearman´s test and corrected for multiple comparisons using the FDR method. The Kaplan–Meier survival analyses were drawn to evaluate the prognostic value of the SPIN1 related DEGs using the R packages “survival”. And survival analyses were conducted with stratification based on gender and M stage classification. Specially, the survfit function was used to construct a survival model based on the formula Surv (time = OS.time, event = OS) ∼ group, where OS.time was the survival time variable, OS was the survival status variable, and group was the grouping variable. Finally, the ggsurvplot function was used to visualize the results of the survival model.

## 3 Results

### 3.1 Correlation of SPIN1 expression with clinical factors and prognosis in GC

**FIGURE 1:**
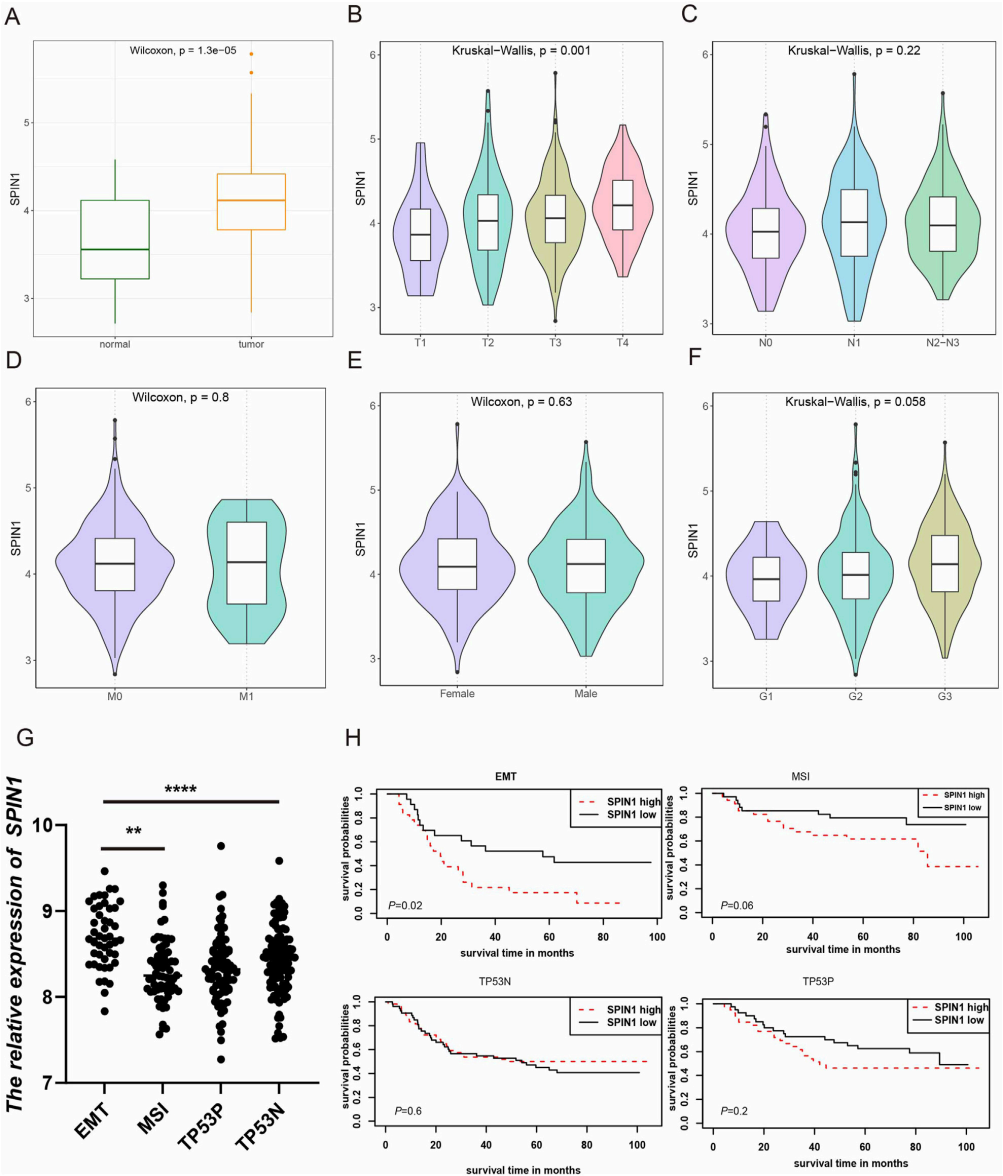
Correlation of SPIN1 expression with clinical factors in GC. **(A)** SPIN1 was more highly expressed in GC compared with normal tissues. **(B)** SPIN1 expression was significantly associated with T stage of GC patients. **(C–F)** SPIN1 expression had no relationship with other clinicopathological features of GC patients, such as gender, M stage, N stage and tumor grade. **(G)** SPIN1 exhibits the highest expression levels in the MSS/EMT subtype. **(H)** Elevated expression of SPIN1 is significantly correlated with adverse outcomes in the MSS/EMT subtype, whereas no significant correlation is observed between its expression and the prognosis of the other GC subtypes.

We used TCGA-STAD database to explore the expression levels of SPIN1 in normal and cancer tissues. The findings indicated that SPIN1 expression was significantly elevated in GC tissues compared to normal tissues (p < 0.001), as shown in Figure 1A. We further explored the relationship between SPIN1 expression and the clinicopathological factors of GC patients. As shown in Figure 1B, SPIN1 expression was significantly associated with T stage of GC patients (p < 0.05), while had no relationships with other clinicopathological features, including gender, M stage, N stage and tumor grade (Figures 1C–F).

In addition, we identified correlations between SPIN1 expression and the four molecular subtypes proposed by the ACRG cohort (Cristescu et al., 2015), as well as its association with prognosis. Our analysis revealed that SPIN1 exhibits the highest expression levels in the MSS/EMT subtype, which is associated with the poorest prognosis among the four subtypes (Figure 1G). Importantly, elevated SPIN1expression is significantly correlated with adverse outcomes in the MSS/EMT subtype, while no significant correlation is observed between SPIN1 expression and prognosis in the other GC subtypes (Figure 1H).

Based on the ACRG dataset, univariate and multivariate regression analyses were performed to evaluate potential prognostic factors. In the univariate analysis, SPIN1 expression (HR = 1.42, 95% CI: 1.03–1.96, p = 0.032) demonstrated significant associations with overall survival. Subsequent multivariate regression analysis confirmed that SPIN1 expression (HR = 1.41, 95% CI: 1.02–1.96, p = 0.039) remained independent predictors of poor survival outcomes. Statistical significance was defined as p < 0.05. These results highlight the critical roles of SPIN1 expression in prognostic stratification within the ACRG cohort (Supplementary Figure S1).

### 3.2 Correlation analysis of SPIN1 with cancer cell proliferation and metastasis

**FIGURE 2:**
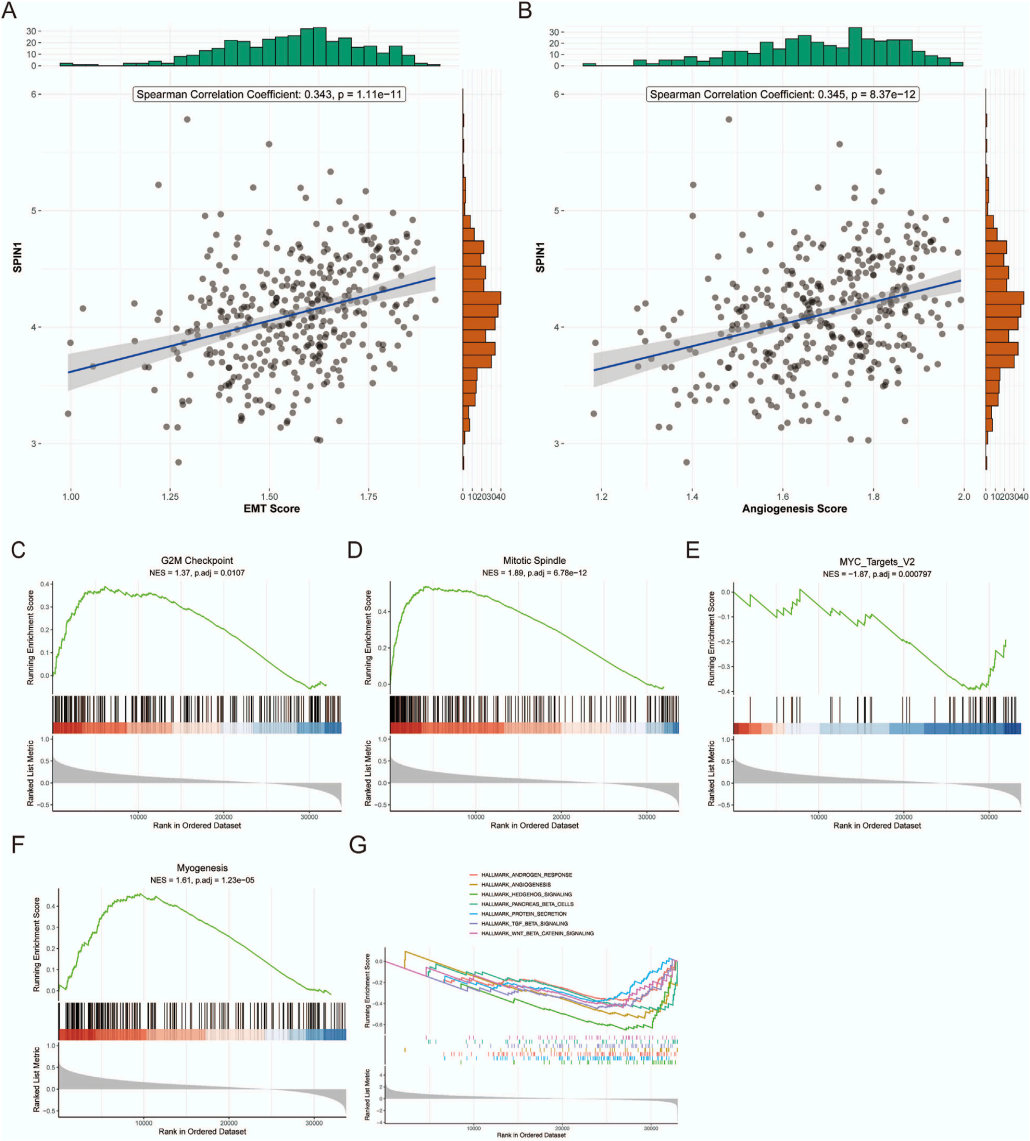
Correlation analysis of SPIN1 with cancer cell proliferation and metastasis. **(A,B)** SPIN1 expression was positively correlated with EMT score and angiogenesis score. **(C–F)**. SPIN1 expression strongly correlated with all four of the cell proliferation-related gene sets in the Hallmark collection (G2M Checkpoint, MYC Targets v2, Myogenesis and Mitotic Spindle) **(G)** Gene Set Enrichment Analysis was performed on SPIN1-high expressing tumors.

We investigated the relationship between SPIN1 expression and cancer cell metastasis, including EMT score and angiogenesis score. The results showed that SPIN1 expression was positively correlated with EMT score and angiogenesis score (Figures 2A,B). Then we evaluated the SPIN1 expression and cancer cell proliferation. SPIN1 expression strongly correlated with all four of the cell proliferation-related gene sets in the Hallmark collection (G2M Checkpoint, MYC Targets v2, Myogenesis and Mitotic Spindle) (Figures 2C–F).

In addition, the analysis dataset was partitioned into two subgroups - SPIN1-high and SPIN1-low - with the median expression value serving as the grouping threshold. The high expression of SPIN1 was significantly enriched in the ANDROGEN_RESPONSE, ANGIOGENESIS, and WNT_BETA_CATENIN_SIGNALING pathways, among others (Figure 2G). The GSVA -based gene set enrichment results demonstrated that the SPIN1 high-expression subgroups in both TCGA and ACRG datasets were significantly enriched in pathways such as ADHERENS_JUNCTION, WNT_SIGNALING_PATHWAY, and TGF_BETA_SIGNALING_ PATHWAY. These findings suggest a potential mechanistic link between elevated SPIN1 expression and dysregulation of cell adhesion, Wnt signaling, and TGF-β-mediated processes in GC (Supplementary Figure S2).

### 3.3 Comparison of mutations in high and low SPIN1 expression groups

**FIGURE 3:**
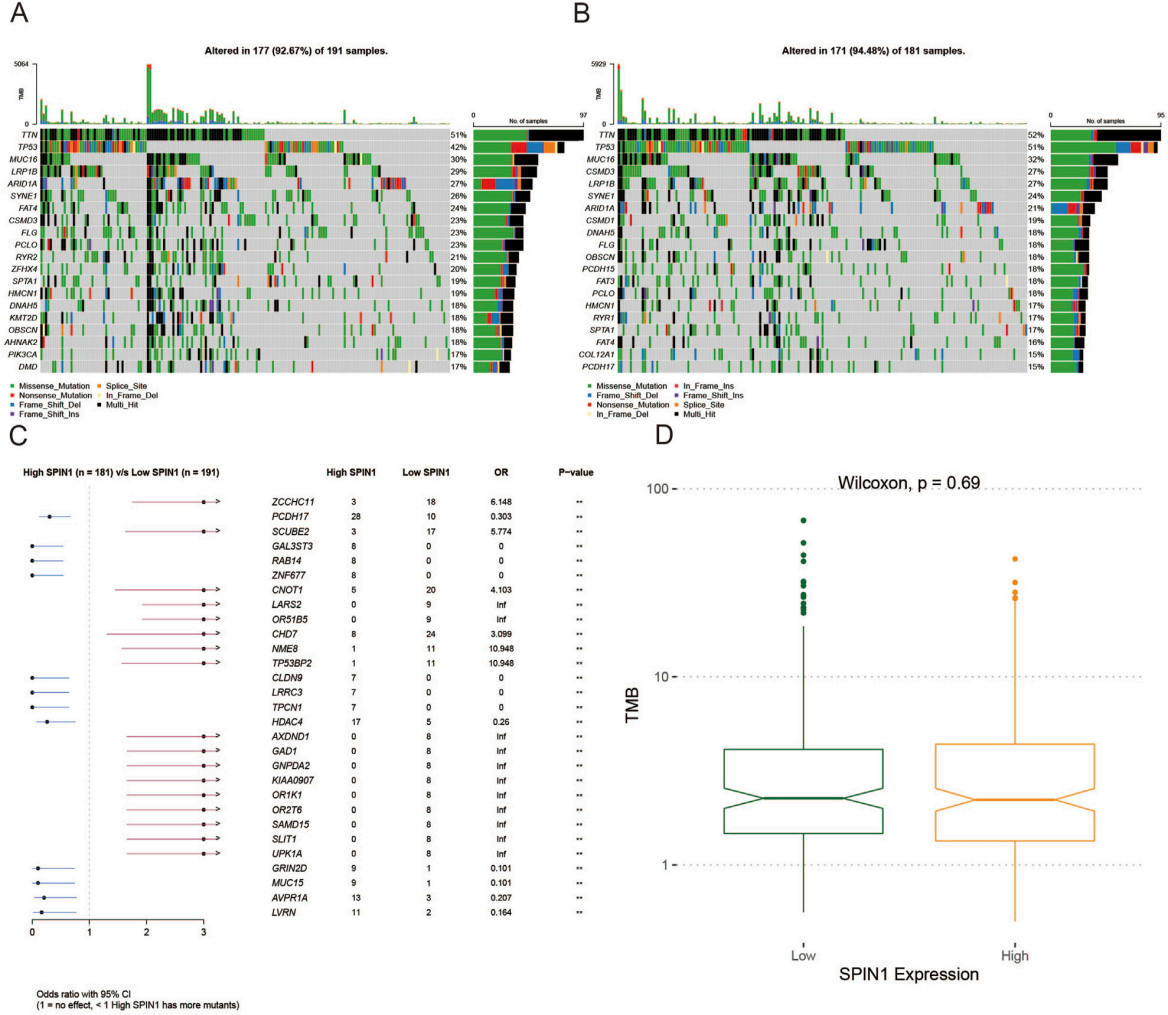
Comparison of mutations in high and low SPIN1 expression groups. **(A)** Among 181 TCGA-STAD samples in high SPIN1 expression group, the mutation frequency of 12 genes was above 20%. **(B)** Among 191 TCGA-STAD samples in low SPIN1 expression group, the mutation frequency of 7 genes was above 18%. **(C)** ZCCHC11 with the largest mutation difference between SPIN1 high and low groups, followed by PCDH17 and SCUBE2. **(D)** There was no significant difference in TMB scores between the SPIN1 high and low expression groups.

Among 181 TCGA-STAD samples in high SPIN1 expression group, the mutation frequency of 12 genes was above 20% (Figure 3A). Among 191 TCGA-STAD samples in low SPIN1 expression group, the mutation frequency of 7 genes was above 18% (Figure 3B). It was found that TTN exhibited the highest mutation frequency both in high and low SPIN1 expression groups, followed by TP53 and MUC16. Then we compared the mutant genes in the SPIN1 high and low expression groups, and ZCCHC11 with the largest mutation difference between SPIN1 high and low groups, followed by PCDH17 and SCUBE2 (Figure 3C). Finally, there was no significant difference in TMB scores between the SPIN1 high and low expression groups (Figure 3D).

### 3.4 Evaluation of the immune microenvironment landscape and drug sensitivity analysis

**FIGURE 4:**
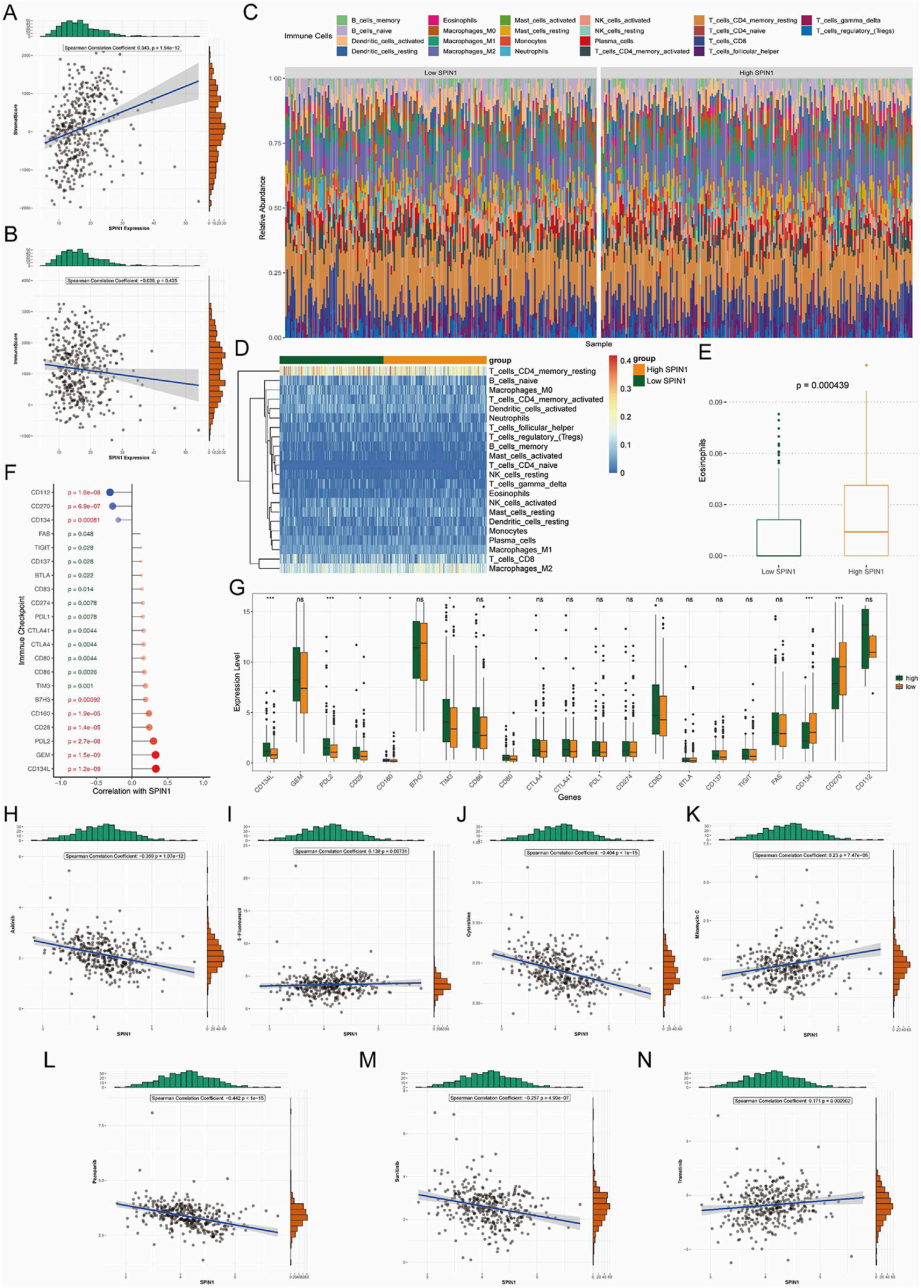
Evaluation of the immune microenvironment landscape and drug sensitivity analysis. **(A)** The stromal score had the positive correlation with SPIN1 expression (cor = 0.343, p < 0.001). **(B)** The correlation between SPIN1 expression and immune score was not significant. **(C)** CIBERSORT databases were used to screen the differential immune cell between the high and low SPIN1 expression groups. Using the par function, the immune cell percentage in each GC sample was calculated and the stacked histogram was plotted. **(D)** The difference of immune cell infiltration pattern between SPIN1 high expression group and SPIN1 low expression group were not significant. **(E)** The number of eosinophils is significantly higher in the high SPIN1 expression group compared to the low SPIN1 expression group. **(F)** The expressions of 21 immune checkpoints were significantly correlated with SPIN1 expression. Among them, CD134L had the strong positive correlation with SPIN1. CD112 and SPIN1 had the strongest negative correlation. **(G)** Analysis of immune checkpoint expression. **(H–N)** The half-maximal inhibitory concentration (IC50) was used to predict the treatment response to 7 drugs in TCGA-STAD cohort. The result of the correlation between IC50 and SPIN1 expression showed that Mitomycin C had the strongest positive correlation to SPIN1, Pazopanib had the strongest negative correlation to SPIN1.

We calculated immune/stromal scores and their correlation with SPIN1 expression. The results showed that the stromal score had the positive correlation with SPIN1 expression (cor = 0.343, p < 0.001) (Figure 4A). The correlation between SPIN1 expression and immune score was not significant (Figure 4B). Then we used CIBERSORT databases to screen the differential immune cell between the high and low SPIN1 expression groups. Using the par function, the immune cell percentage in each GC sample was calculated and the stacked histogram was plotted (Figure 4C). The difference of immune cell infiltration pattern between SPIN1 high expression group and SPIN1 low expression group were not significant (Figure 4D). Among them, we obtained 1 differential immune cell called eosinophils (Figure 4E).

Furthermore, we calculated the correlations among the SPIN1 expression and 37 immune checkpoints. The results indicated that the expressions of 21 immune checkpoints were significantly correlated with SPIN1 expression. Among them, CD134L had the strong positive correlation with SPIN1 (cor = 0.335), the next was GEM (cor = 0.33). CD112 and SPIN1 had the strongest negative correlation (cor = −0.309), followed by the correlation between CD270 and SPIN1(cor = −0.272) (Figure 4F). Analysis of immune checkpoint expression revealed that, compared to the SPIN1-low group, the SPIN1-high group exhibited significantly upregulated levels of CD134L, PD-L2, and CD28, while CD134 and CD270 were markedly downregulated (Figure 4G). We explored the relationship between SPIN1 expression and chemoresistance. The half-maximal inhibitory concentration (IC50) was used to predict the treatment response to 7 drugs in TCGA-STAD cohort (Figures 4H–N). The result of the correlation between IC50 and SPIN1 expression showed that Mitomycin C had the strongest positive correlation to SPIN1, high SPIN1 expression samples were more sensitive to Mitomycin C, Trametinib, 5-Fluorouracil (p < 0.05). Pazopanib had the strongest negative correlation to SPIN1, low SPIN1 expression samples were more sensitive to Axitinib, Cytarabine, Pazopanib and Sunitinib.

### 3.5 Correlations of hub genes with m6A and m5C associated genes

**FIGURE 5:**
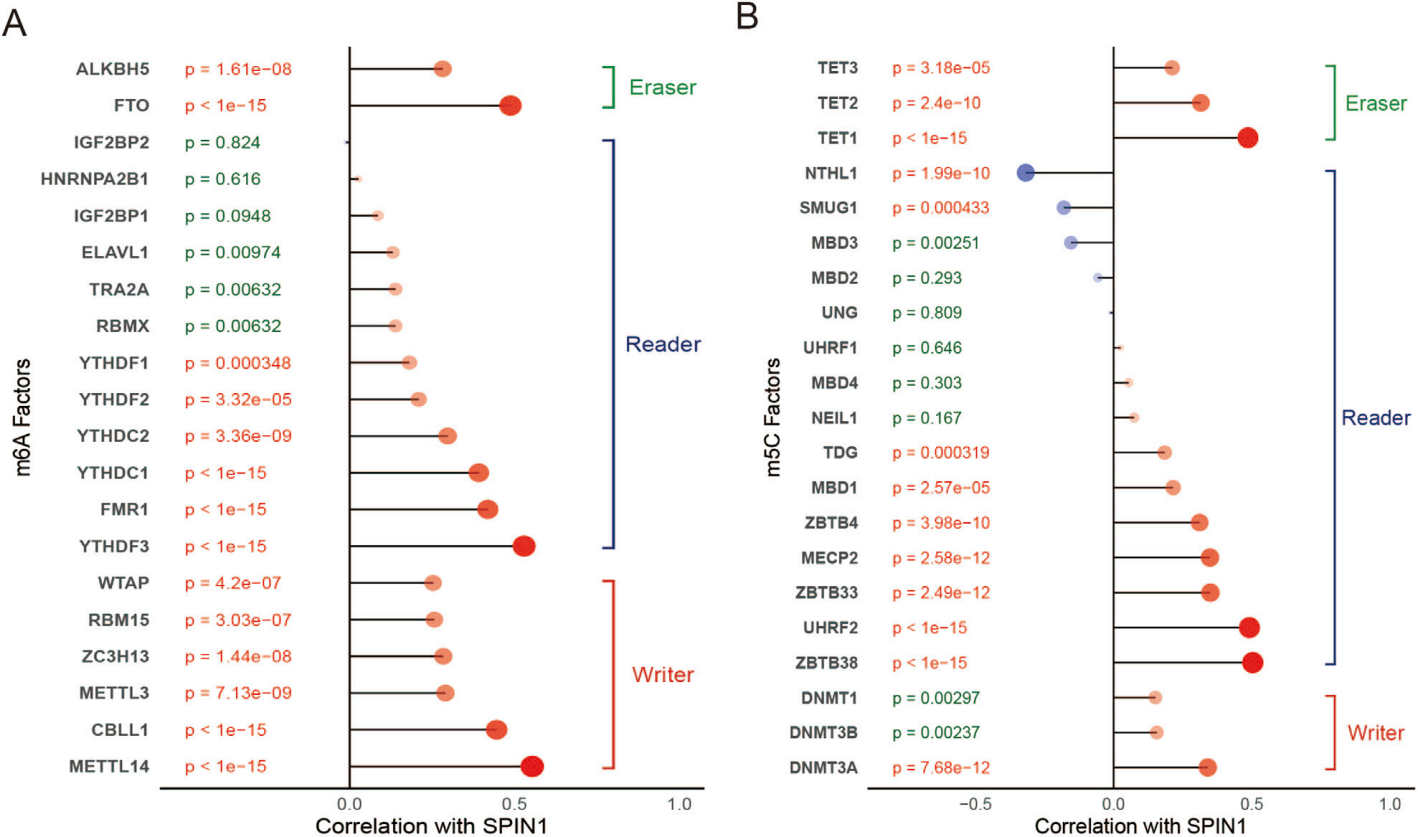
Correlations of hub genes with m6A and m5C associated genes. **(A)** We constructed the correlation analysis between the expression of SPIN1 and 20 m6A regulators, the results showed that most m6A regulators were positively associated with SPIN1, except for IGF2BP2. METTL14 was the most positively correlated with SPIN1, the next was YTHDF3. **(B)** We detected the correlation analysis between SPIN1 and 21 m5C regulators, ZBTB38 was the most positively correlated with SPIN1, the next was UHRF2. NTHL2 had the strongest negative correlation to SPIN1, followed by SMUG1.

Accumulating evidence suggests that DNA methylation alterations play a significant role in tumorigenesis on aspects of cell proliferation, differentiation and pharmacoresistance. Therefore, we tried to analyze crosstalk between m6A and 5 mC regulators on expression of SPIN1 in GC. We constructed the correlation analysis between the expression of SPIN1 and 20 m6A regulators (Figure 5A), and we found most m6A regulators were positively associated with SPIN1, except for IGF2BP2(cor = −0.01). METTL14 was the most positively correlated with SPIN1(cor = 0.553), the next was YTHDF3 (cor = 0.529). Then we evaluated the expression of 21 m5C-related genes in GC. We detected the correlation analysis between SPIN1 and 21 m5C regulators (Figure 5B), and we found ZBTB38 was the most positively correlated with SPIN1(cor = 0.503), the next was UHRF2(cor = 0.492). NTHL1 had the strongest negative correlation to SPIN1(cor = −0.318), followed by SMUG1 (cor = −0.180). In addition, SPIN1-high tumors exhibited significantly upregulated expression of RNA modification writers METTL3 and NSUN2 (Supplementary Figure S3).

### 3.6 Identification of DEGs related to SPIN1

**FIGURE 6:**
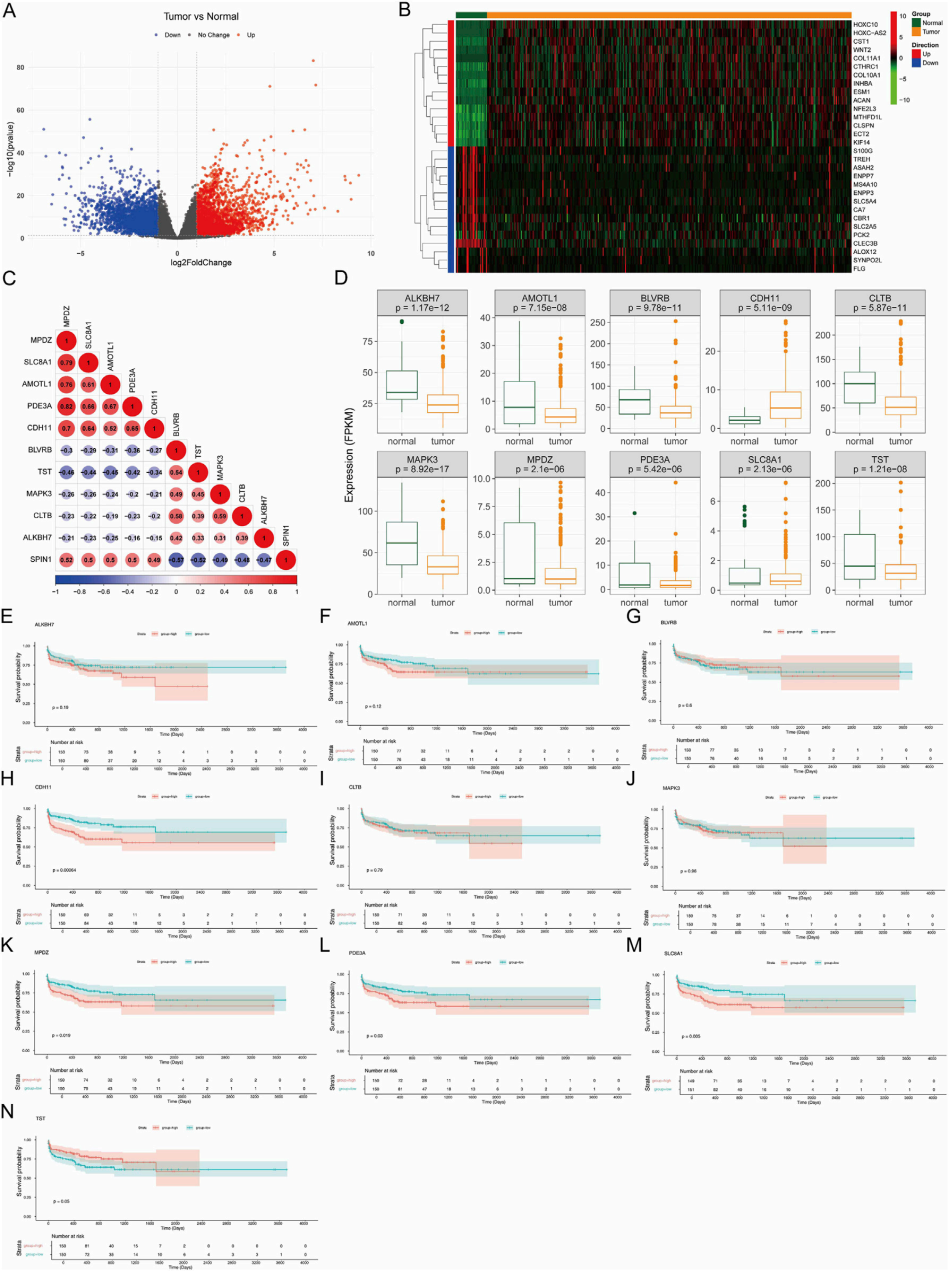
Identification of DEGs related to SPIN1. **(A)** By comparing tumor and normal tissue samples, a total of 7097 DEGs including 4133 upregulated genes and 2964 downregulated genes were detected. **(B)** The expression of top 15 upregulated and downregulated DEGs by heatmap was showed. **(C)** The top 5 DEGs with the most positive and negative correlation to SPIN1 were obtained by Spearman correlation analysis, including MPDZ, SLC8A1, AMOTL1, PDE3A, CDH11, BLVRB, TST, MAPK3, CLTB and ALKBH7. **(D)** The expression of 9 SPIN1-related DEGs were downregulated in tumor sample except CDH11. **(E–N)** Kaplan-Meier survival analysis.

By comparing tumor and normal tissue samples, a total of 7097 DEGs including 4133 upregulated genes and 2964 downregulated genes were detected as shown in Figure 6A. Figure 6B showed the expression of top 15 upregulated and downregulated DEGs by heatmap. After that, the top 5 DEGs with the most positive and negative correlation to SPIN1 were obtained by Spearman correlation analysis, including MPDZ, SLC8A1, AMOTL1, PDE3A, CDH11, BLVRB, TST, MAPK3, CLTB and ALKBH7(Figure 6C). MPDZ had the most positive correlation to SPIN1(cor = 0.52), BLVRB had the most negative correlation to SPIN1(cor = −0.57). Then we analyzed the expression level of 10 SPIN1-related DEGs. The results showed that the expression of 9 SPIN1-related DEGs were downregulated in tumor sample except CDH11 (Figure 6D). We then screened SPIN1-related DEGs which associated with prognosis, Kaplan-Meier survival analysis demonstrated that CDH11, MPDZ, PDE3A and SLC8A1 were associated with the prognosis of GC (Figures 6E–N).

## 4 Discussion

Gastric cancer (GC) ranks among the most prevalent malignant neoplasms and is a leading cause of cancer-related mortality globally, thereby representing a substantial threat to public health. Although SPIN1 has been implicated in tumorigenesis and tumor progression, the underlying molecular mechanisms remain inadequately elucidated. In this study, we conducted a comprehensive analysis of the associations between SPIN1 expression and various factors, including tumor progression, clinical stage, immune microenvironment, drug sensitivity and epigenetic regulation in a cohort of 375 GC patients and 32 controls. Furthermore, genes exhibiting a strong correlation with SPIN1 were identified through Spearman correlation analysis.

The Asian Cancer Research Group’s molecular classification has been pivotal in highlighting GC heterogeneity, identifying four distinct subtypes with unique characteristics and prognostic implications. Notably, SPIN1 expression is highest in the MSS/EMT subtype, known for its aggressive nature and therapy resistance, suggesting a potential link between SPIN1 and this aggressive phenotype. The association of high SPIN1 expression with poor outcomes underscores the need for novel treatments for the MSS/EMT subtype. Conversely, no significant correlation between SPIN1 expression and prognosis was found in the other GC subtypes, indicating a subtype-specific role for SPIN1 and emphasizing the importance of personalized medicine in GC treatment.

Furthermore, our analysis revealed a positive correlation between SPIN1 expression and the EMT score, angiogenesis score, as well as gene sets associated with cell proliferation. These findings indicate a significant association between SPIN1 and both metastasis and cell proliferation in GC, corroborating our prior results (Lv et al., 2020). Furthermore, SPIN1 overexpression enriches pathways such as **ANDROGEN RESPONSE**, **ANGIOGENESIS**, and **WNT/Β-CATENIN SIGNALING**. The **ANDROGEN RESPONSE** pathway plays a pivotal role in prostate cancer initiation, progression, and the development of castration-resistant prostate cancer (CRPC) (Rhee et al., 2024). Tumor growth and metastasis rely on **Angiogenesis** to supply oxygen and nutrients (Lugano et al., 2020). In colorectal cancer, SPIN1 promotes tumor cell proliferation and invasion by activating the **Wnt/β-catenin signaling pathway** (Zhou et al., 2021). Collectively, these findings suggest that SPIN1 may drive tumorigenesis and progression through these interconnected pathways.

TTN demonstrated the highest mutation frequency in both high and low SPIN1 expression groups, followed by TP53 and MUC16. Notably, ZCCHC11 exhibited the most significant difference in mutation frequency between the high and low SPIN1 expression groups. Mutations in MUC16 and TTN were associated with associated with improved overall survival (OS), and the mutation status and number of mutations in MUC16 and TTN were effective predictors of tumor mutational burden (TMB) (Yang et al., 2020). Gaza et al. have elucidated the carcinogenic role of ZCCHC11 in liver cancer (Gaza et al., 2021). To the best of our knowledge, no studies have yet investigated the role of ZCCHC11 in GC. Hence, the potential synergistic oncogenic effect of ZCCHC11 and SPINI in GC is an area we intend to explore in future studies. Therefore, we aim to explore the potential synergistic oncogenic effects of ZCCHC11 and SPIN1 in GC in future research endeavors.

Recent studies have elucidated a significant correlation between the tumor immune microenvironment and the malignant progression of cancer. In this research, we conducted an analysis of the differential infiltration of immune cells in GC by comparing groups with high and low SPIN1 expression. Our findings identified eosinophils as a differentially infiltrated immune cell type. Eosinophils, known as crucial effector cells in allergic diseases, hold potential therapeutic implications. Emerging evidence indicates that eosinophilic infiltrations occur in various tumors, where they may directly interact with tumor cells or indirectly influence tumor progression by modulating the tumor microenvironment (TME) (Grisaru-Tal et al., 2020). Caruso et al. (Caruso et al., 2002) reported on tumor-associated eosinophils in human GC, noting that tumor cells in proximity to eosinophils exhibited signs of autophagic cell death. These findings underscore the importance of eosinophils as accessory cells in cancer immunotherapy and highlight their potential as targets for future immune checkpoint blockade therapies. To our knowledge, no prior studies have examined the association between SPIN1 expression and both the immune microenvironment and eosinophils, highlighting a promising area for future research.

SPIN1 has been reported to be associated with radiotherapy sensitivity (Jin et al., 2021; Yu et al., 2022; Chen et al., 2019) as well as chemotherapy resistance (Chen X. et al., 2018; Chen et al., 2016) in previous studies. However, the relationship between SPIN1 expression and chemoresistance in GC remains unclear. Our correlation analysis demonstrated that Mitomycin C, Trametinib and 5-Fluorouracil positively correlate with SPIN1 expression, whereas Pazopanib exhibits the strongest negative correlation. It is noteworthy that Mitomycin C, an antibiotic antineoplastic agent, is widely utilized in the treatment of GC. The observed resistance to Mitomycin C, a DNA crosslinking agent, may be attributed to SPIN1-mediated activation of the G2/M checkpoint pathway, which facilitates DNA damage repair and cell survival (Nakayama et al., 2020). Similarly, resistance to Trametinib likely involves dual mechanisms: (1) dysregulation of G2/M checkpoint functionality and (2) modulation of c-MYC-dependent MYC Targets v2 pathway activity (Silvis et al., 2023; Cheng et al., 2024). These findings collectively suggest that SPIN1-driven chemoresistance may arise from its regulatory roles in cell cycle progression and proliferative signaling pathways.

Therapeutically, targeting downstream effectors of SPIN1, such as FOXM1 or MDM2 inhibitors, represents a promising strategy to overcome resistance. Mechanistically, SPIN1 activates FOXM1 by promoting MDM2-mediated ubiquitination and degradation of FOXO3a, thereby establishing the SPIN1-MDM2-FOXO3a/FOXM1 signaling axis. This axis has been implicated in non-small cell lung cancer progression and radiation resistance (Zhong et al., 2024), highlighting its potential as a therapeutic vulnerability in SPIN1-high malignancies. Moreover, samples exhibiting low SPIN1 expression showed heightened sensitivity to Axitinib, Cytarabine, Pazopanib, and Sunitinib. The combination therapy of mitomycin C with cisplatin, 5-fluorouracil and leucovorin has been validated as both safe and effective for the treatment of advanced GC (Cascinu et al., 2002). Pazopanib, an oral tyrosine kinase inhibitor, is recognized for its selective inhibition of the VEGFR-1, -2, -3, c-kit, and PDGF-R pathways, thereby impeding angiogenesis.

The advent of immune checkpoint inhibitors marks a pivotal advancement in cancer immunotherapy research. Nonetheless, it is essential to recognize that some patients may not experience the full therapeutic benefits of immunotherapy due to intrinsic or acquired drug resistance. As a result, there is an increasing necessity for oncologists to investigate novel immune checkpoint inhibitors. In this context, Kovács et al. have developed a comprehensive database and web platform designed to investigating biomarkers associated with responses to immunotherapy. Their findings identify SPIN1 as a particularly promising druggable gene candidate linked to resistance against anti-PD-1 therapy (Kovács et al., 2023). Building upon their research, our study aims to evaluate the immune microenvironment landscape and its correlation with SPIN1 expression. Our results indicate that 21 immune checkpoints are associated with SPIN1 expression, with CD134L showing a positive correlation and CD112 demonstrating a negative correlation with SPIN1 levels. Elevated SPIN1 expression may enhance immune checkpoint inhibition by upregulating PD-L2 expression. The subsequent PD-L1/PD-2 interaction suppresses T-cell activation and proliferation, thereby promoting immune escape (Dutta et al., 2023). These findings offer valuable insights into novel strategies for leveraging SPIN1 in cancer immunotherapy applications. Future research will focus on investigating the role of SPIN1 within immune cells and elucidating its influence on the expression and activity of immune checkpoints. Additionally, we aim to validate CD134L and CD112 as potential biomarkers for predicting responses to immunotherapy, while also investigating their specific mechanisms of interaction with SPIN1.

N6-methyladenosine (m6A) and 5-methylcytidine (m5C) represent two methylation modifications present on RNA. Aberrant alterations in key genes involved in m6A and m5C RNA methylation may significantly influence the development and prognosis of GC (Jing et al., 2021; Fang et al., 2023). We investigated the interplay between m6A and m5C regulators and SPIN1 expression in GC. Most m6A regulators demonstrated a positive correlation with SPIN1, with METTL14 exhibiting the strongest association, followed by YTHDF3. METTL14 is a critical component of the m6A methyltransferase complex (MTC), playing a vital role in maintaining appropriate levels of m6A methylation on target genes. In most tumors, METTL14 functions as a tumor suppressor by reducing m6A levels within cancer cells through its activity as an m6A methyltransferase, thereby inhibiting tumor development and progression. However, there are instances where METTL14 has been shown to promote tumor growth (Wang M. et al., 2020). Recent studies in GC have indicated that METTL14 acts as a tumor suppressor gene (Fan et al., 2022; Ge et al., 2023; Liu et al., 2021). This observation contradicts the established biological function of SPIN1 in GC, necessitating further investigation into the potential correlation between SPIN1 and METTL14 in GC, as well as their underlying mechanisms. YTHDF3, an m6A reader protein, is known to contributes to the development and progression of various cancers including GC (Shi et al., 2022; Lin et al., 2022; Chang et al., 2020; Yu et al., 2023). Furthermore, this study demonstrated that METTL3 expression was significantly upregulated in tumors with high SPIN1 levels. Mechanistically, METTL3 enhances the translation efficiency of Jak1 mRNA through m6A modification, thereby activating the JAK1-STAT3 signaling pathway and facilitating the formation of an immunosuppressive tumor microenvironment (Xiong et al., 2022). These findings suggest that SPIN1 may participate in post-transcriptional gene regulation by modulating METTL3-mediated m6A modification, consequently influencing tumor biological behaviors.

Among m5C regulators, ZBTB38 exhibited the most significant positive correlation with SPIN1, followed by UHRF2. In contrast, NTHL2 showed the strongest negative correlation with SPIN1, with SMUG1 being the next most negatively correlated. Although ZBTB38 has not been previously investigated in GC, it has been characterized as a tumor suppressor in prostate cancer (Ding et al., 2021; de Dieuleveult et al., 2020) and conversely, as an oncogene in bladder cancer (Jing et al., 2019). UHRF2 is known for its role as a transcriptional co-regulator in the epithelial-mesenchymal transition (EMT) process within GC (Lai et al., 2016), a function that aligns with the role of SPIN1. Furthermore, this study revealed that NSUN2 expression was significantly upregulated in tumors with elevated SPIN1 levels. Previous studies have demonstrated that NSUN2 suppresses ferroptosis and promotes EC progression by mediating m5C modification of SLC7A11 mRNA, which facilitates YBX1 recruitment to enhance transcript stability (Chen et al., 2024). These findings suggest that SPIN1 may modulate post-transcriptional gene regulation through NSUN2-dependent m5C modification, consequently influencing tumor biological behaviors. To date, no correlations between SPIN1 and the aforementioned regulatory factors have been identified in the literature. Future studies are warranted to further delineate the correlation and underlying mechanisms of YTHDF3, UHRF2, and SPIN1 within GC cell lines and tissue samples.

Through our screening, we screened 10 DEGs with the strongest correlation with SPIN1 in GC, among which CDH11, MPDZ, PDE3A, and SLC8A1 may serve as potential prognostic biomarkers in GC. However, these findings currently rely solely on bioinformatics analyses, further experimental validation is warranted to confirm their clinical applicability. Besides, prior research has also indicated that elevated CDH11 expression is associated with distant metastasis and a poorer prognosis in GC (Mita et al., 2023; Wang Q. et al., 2020; Chen PF. et al., 2018). Subsequent research will focus on the interplay between SPIN1 and CDH11 in GC, with the objective of investigating the potential synergistic effects that may contribute to the progression.

Furthermore, the present study possesses several limitations that warrant consideration. Firstly, the marked sample size disparity between the control group (32 cases) and gastric cancer cohort (375 cases) may introduce statistical bias. To address this limitation, we plan to expand our cohort in subsequent investigations to further validate the functional role of SPIN1 in gastric carcinogenesis. Secondly, although computational analyses have provided valuable preliminary insights, these findings remain at the hypothesis-generating stage without experimental validation. Consequently, we intend to undertake systematic validation through *in vitro* and *in vivo* models to rigorously assess the reliability and translational potential of these discoveries.

## 5 Conclusion

In conclusion, this preliminary bioinformatics analysis suggests that SPIN1 may play a potential role in GC progression, with our computational models indicating possible molecular associations that warrant further investigation. Furthermore, the findings highlight the necessity for further research to unravel the complex molecular pathways involving SPIN1 in GC and to understand its interactions with other biomarkers.

## Data Availability

The original contributions presented in the study are included in the article/Supplementary Material, further inquiries can be directed to the corresponding author.

## Supplementary material

SUPPLEMENTARY FIGURE S1 Univariate and multivariate Cox regression analysis.

SUPPLEMENTARY FIGURE S2 GSVA enrichment analysis based on the TCGA and ACRG datasets.

SUPPLEMENTARY FIGURE S3 Expression level analysis of METTL3 and NSUN2.
