# Supplementary figures and images for: Gene expression profiling of SPIN1 in gastric cancer: insights into tumorigenesis and potential therapeutic targets
Source: Front Genet. 2025 Jun 11;16:1510849. doi: 10.3389/fgene.2025.1510849 (PMC12188309; doi:10.3389/fgene.2025.1510849)

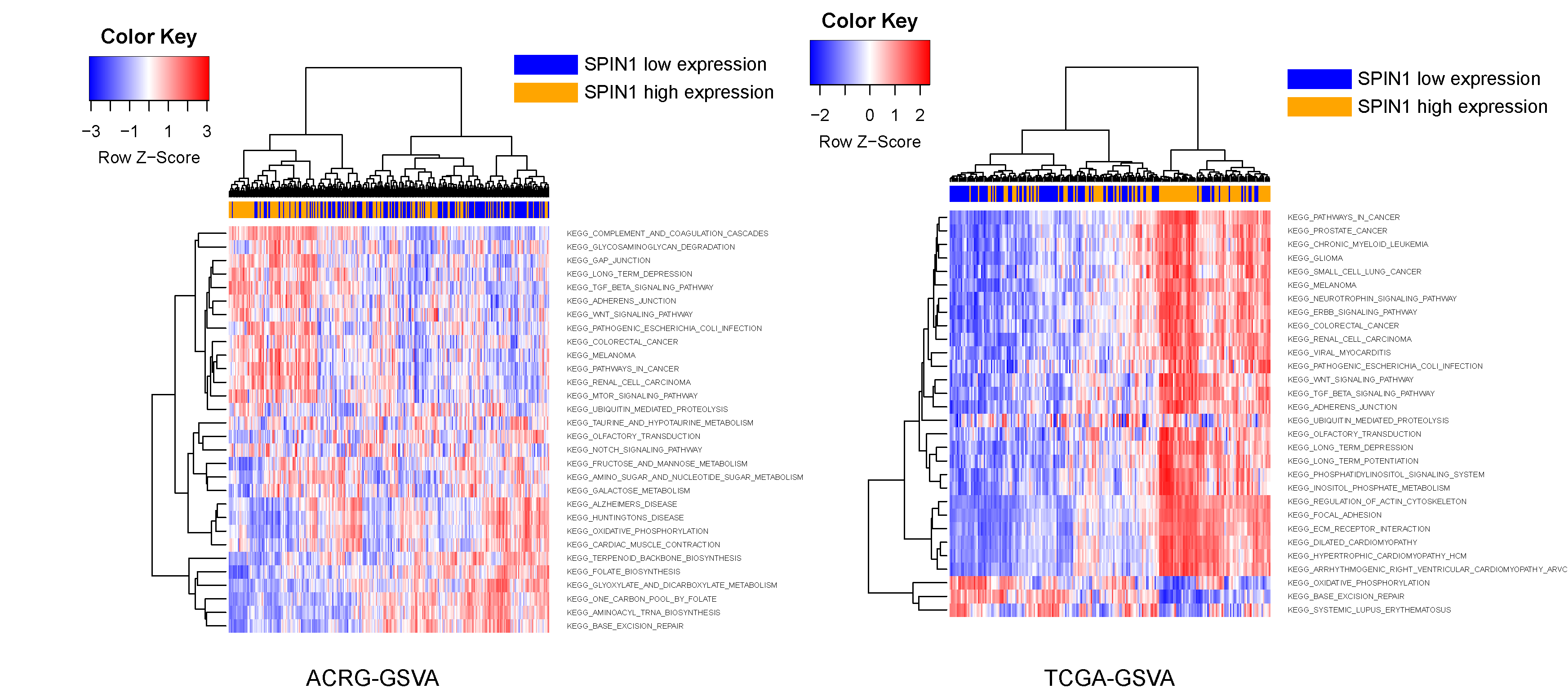

Supplement: Supplementary file 2 [file Image2.tif]

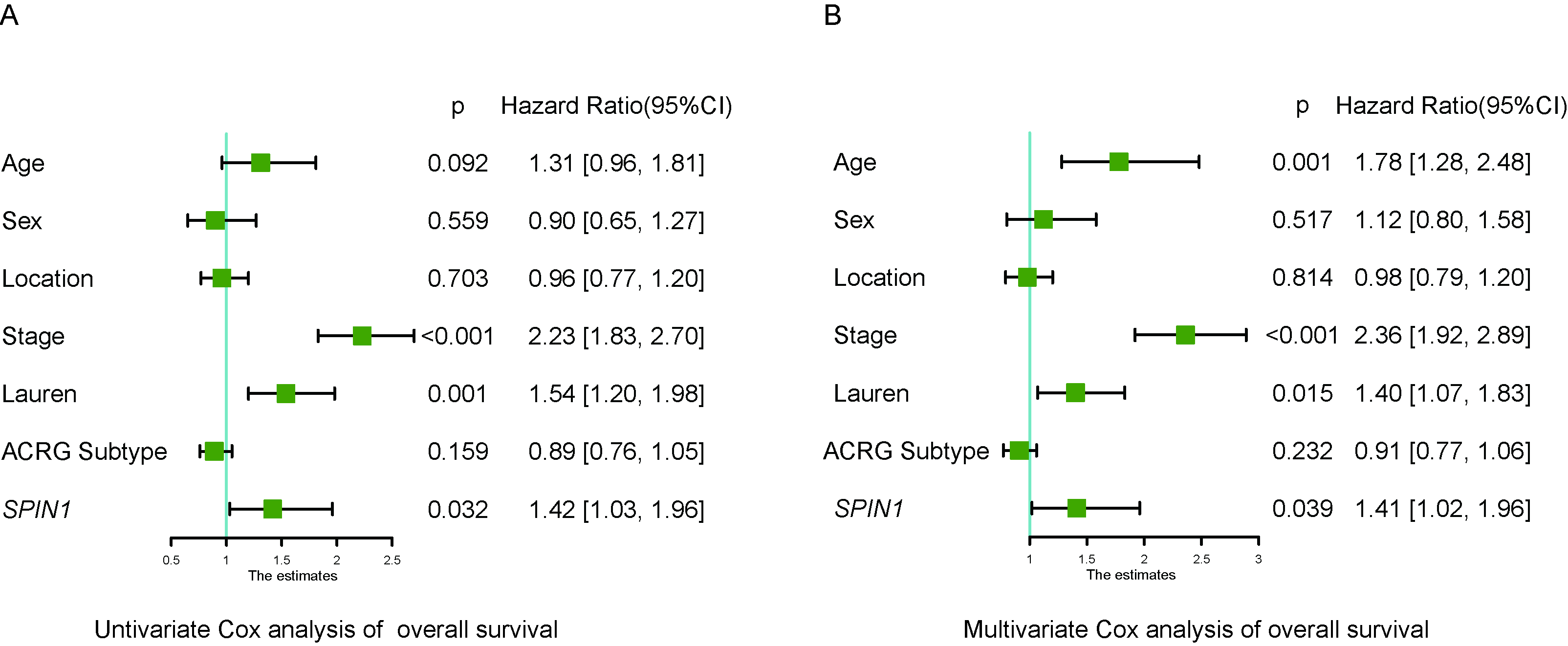

Supplement: Supplementary file 3 [file Image1.tif]
